# The *Lotus japonicus NPF3.1* Is a Nodule-Induced Gene That Plays a Positive Role in Nodule Functioning

**DOI:** 10.3389/fpls.2021.688187

**Published:** 2021-06-18

**Authors:** Ylenia Vittozzi, Marcin Nadzieja, Alessandra Rogato, Simona Radutoiu, Vladimir Totev Valkov, Maurizio Chiurazzi

**Affiliations:** ^1^Institute of Biosciences and Bioresources (IBBR), Italian National Research Council (CNR), Napoli, Italy; ^2^Department of Molecular Biology and Genetics, Aarhus University, Aarhus, Denmark

**Keywords:** nitrate transport, N_2_-fixation, nodule, insertion mutants, *Lotus japonicus*

## Abstract

Nitrogen-fixing nodules are new organs formed on legume roots as a result of the beneficial interaction with the soil bacteria, rhizobia. Proteins of the nitrate transporter 1/peptide transporter family (NPF) are largely represented in the subcategory of nodule-induced transporters identified in mature nodules. The role of nitrate as a signal/nutrient regulating nodule functioning has been recently highlighted in the literature, and NPFs may play a central role in both the permissive and inhibitory pathways controlling N_2_-fixation efficiency. In this study, we present the characterization of the *Lotus japonicus* LjNPF3.1 gene. LjNPF3.1 is upregulated in mature nodules. Promoter studies show transcriptional activation confined to the cortical region of both roots and nodules. Under symbiotic conditions, Ljnpf3.1-knockout mutant’s display reduced shoot development and anthocyanin accumulation as a result of nutrient deprivation. Altogether, LjNPF3.1 plays a role in maximizing the beneficial outcome of the root nodule symbiosis.

## Introduction

As sessile organisms, land plants developed mechanisms that enable them to cope with the dynamically changing availability of nutrients in the soil (Williams and Miller, 2001; Rogato et al., 2010). Nitrogen in the form of nitrates is often a limiting resource for supporting plant growth in temperate climates (Miller and Cramer, 2005). A primary role in the network governing nitrate uptake, assimilation, storage, and distribution among different plant tissues and organs is played by two protein families, the low-affinity nitrate transporter peptide family (NPF; LATS > 0.5 mM) and the high-affinity nitrate transporter system (NRT2; HATS < 0.5 mM). NPF is a large plant family consisting of 53, 93, and 86 members in *Arabidopsis thaliana*, *Oryza sativa*, and *Lotus japonicus*, respectively (Tsay et al., 2007; Léran et al., 2014; Sol et al., 2019). To date, nitrate transport activity has been reported for 17 *A. thaliana* NPF proteins (Corratgé-Faillie and Lacombe, 2017), with AtNPF6.3 being the only exception as this displays a switching dual nitrate affinity, controlled *via* the phosphorylation of the threonine residue at position 101, in response to fluctuating external concentrations (Liu et al., 1999; Liu and Tsay, 2003; Parker and Newstead, 2014; Sun et al., 2014). The extended functional characterization carried out in *A. thaliana* is allowing the unraveling of the network of the *AtNPF* distinct functional roles. A variegated pattern of spatiotemporal *NPF* gene expression through the whole plant body is crucial to provide nitrate uptake from the soil, upward and downward long-distance transport, distribution from source to sink tissues, intercellular flux, and cellular nitrate storage redistribution (Wang et al., 2018). NPF members are also able to transport substrates other than nitrate, including di-/tripeptides, amino acids, glucosinolates, malate, auxin, abscisic acid (ABA), gibberellic acid, and jasmonic acid (Frommer et al., 1994; Jeong et al., 2004; Waterworth and Bray, 2006; Krouk et al., 2010; Kanno et al., 2012; Nour-Eldin et al., 2012; Saito et al., 2015; Tal et al., 2016). The different transport capacities are distributed among the eight NPF subclades identified in plants, and the prediction of the transported substrate cannot be determined from the sequences data alone (Léran et al., 2014). In a few cases, the capability of the NPF members to transport both nitrate and hormones with different affinities has been reported, and this multispecificity could suggest intriguing roles of NPF proteins for the integration of environmental and physiological information linked to the relative availability of different nutrients (Krouk et al., 2010; Kanno et al., 2012; Saito et al., 2015; Corratgé-Faillie and Lacombe, 2017). So far, this role has been demonstrated only for the *AtNPF6.3* gene, which also functions as an auxin transport facilitator to modulate lateral root elongation in response to nitrate (Krouk et al., 2010).

The nodule organogenesis competence evolved in legume plants as a result of the symbiotic interaction with rhizobia partner where an ideal microenvironmental niche enables the conversion of atmospheric N_2_ into the plant assimilable NH_3_. The invaded cells of the N_2_-fixing nodules are filled with symbiosomes, the organelles originated by an endocytosis-like process enclosing invading bacteria in a plant-derived membrane whose formation involves an exocytotic process [peri-bacteroidal membrane (PBM)]. Inside the symbiosome, bacteria stop dividing and differentiate into the N_2_-fixing bacteroids. For the correct function of the nodules, it is crucial to maintain the balance between the microaerophilic condition, which protects nitrogenase from inactivation, and the high rates of respiration taking place in the cytosol and bacteroid compartments of the invaded cells (Bergensen, 1996; Witty and Minchin, 1998). These conflicting demands are met *via* the oxygen barrier constituted by parenchyma cell layers and mainly by the action of the high-affinity O_2_^−^ binding protein leghemoglobin (Lb), present at millimolar concentrations in the N_2_-fixing cells (Appleby, 1984). The nodulation process is an excellent example of adaptation to the changing environment as all the very demanding steps of nodule formation, development, and functioning are quickly inhibited when sufficient amounts of fixed N are readily available in the soil (Carroll and Gresshoff, 1983; Fujikake et al., 2003; Barbulova et al., 2007; Omrane and Chiurazzi, 2009; Naudin et al., 2011; Cabeza et al., 2014). In the case of nodule functioning, the responsiveness to nitrate has been mainly associated with the inhibitory pathway triggering a dramatic decrease in functional leghemoglobin and nitrogenase activity by the exposure of nodulated roots to high nitrate concentrations (5–10 mM; Arrese-Igor et al., 1998; Cabeza et al., 2014). However, a positive action played by low concentrations of nitrate to maintain the energy status required for efficient N_2_-fixation, ensuring the correct nodule functioning, has been also reported (Horchani et al., 2011; Hicri et al., 2015). More recently, a positive role played by both NPF and NRT2 nitrate transporters for satisfying the required nitrate allocation and distribution into the N_2_-fixing zone of the nodules has been proposed (Valkov et al., 2017, 2020; Wang et al., 2020). In particular, *LjNRT2.4* and *MtNPF7.6* seem to be involved in the nitrate-mediated regulation of nodule function by controlling the quick and fine-tuning of nitric oxide (NO) concentration in the nodule-invaded cells in response to rapid changes in O_2_ availability (Valkov et al., 2020; Wang et al., 2020).

In this study, we report the characterization of another member of the *L. japonicus NPF* family that may contribute to the completion of the route of nitrate toward the nodule N_2_-fixing zone required for a correct nodule functioning in the presence of low-permissive external concentrations.

## Materials and Methods

### Plant Material and Growth Conditions

All experiments were carried out with *L. japonicus* ecotype B-129 F14 GIFU (Handberg and Stougaard, 1992; Jiang and Gresshoff, 1997). Plants were cultivated in Petri dishes, in a growth chamber with a light intensity of 200 μmol m^−2^ s^−1^ at 23°C with a 16 h/8 h day/night cycle. Solid growth media had the same composition as B5 medium (Gamborg, 1970), except that (NH_4_)_2_SO_4_ and KNO_3_ were omitted and/or substituted by different concentrations of KNO_3_. KCl was added to the medium to replace the potassium source. The media containing vitamins (Duchefa catalogue G0415) were buffered with 2.5 mM 2-(N-morpholino) ethanesulfonic acid (MES; Duchefa, M1503.0250), and pH was adjusted to 5.7 with KOH.

For the phenotypic characterization at 6 weeks after inoculation, synchronized lotus seedlings were transferred after germination in pots containing lightweight expanded clay aggregate (LECA, Saint-Gobain Weber A/S,^1^ 2–4 mm diameter) and vermiculite size M (Damolin A/S)^2^ in a 4:1 mixture. Plants were supplemented with 60 ml 0.25 × B&D medium supplemented with the required amount of KNO_3_. Plants were incubated at 23°C under a 16-h light/8-h dark cycle.

*Mesorhizobium loti* inoculation was performed as described in Barbulova et al. (2005). For phenotypic comparisons, after germination, unsynchronized seedlings were discarded. The strain R7A was used for the inoculation experiments and was grown in a liquid TYR medium supplemented with rifampicin (20 mg/L).

### *Lotus japonicus* Hairy Root Transformation Procedures

Binary vectors were conjugated into the *Agrobacterium rhizogenes* 15,834 strain (Stougaard et al., 1987). *A. rhizogenes*-mediated *L. japonicus* transformations have been performed as described in Bastianelli et al. (2009). Inoculation of composite plants was described in Santi et al. (2003).

### Protoplast Transformation

Leaf protoplasts were prepared and transformed according to Pedrazzini et al. (1997), using 3-week-old *Nicotiana tabacum* plants. DNA (40 μg of each construct) was introduced into 1 × 10^6^ protoplasts by PEG-mediated transfection. After 16-h incubation in the dark at 25°C, yellow fluorescent protein (YFP) fluorescence in protoplast cells was detected by confocal microscopy.

### LORE1 Lines Isolation

LORE1 lines 300121103 and 30082596 were obtained from the *LORE1* collection (Fukai et al., 2012; Urbanski et al., 2012; Malolepszy et al., 2016). The plants in the segregating populations were genotyped, and the expression of homozygous plants tested with primers is listed in Supplementary Table S1.

### Determination of Acetylene Reduction Activity

Detached roots with a comparable number of nodules were placed in glass vials. The vials were filled with an acetylene – air mixture (C_2_H_2_; air = 1:9 v/v). After 30 min of incubation at 25°C, the amount of ethylene in the gas phase was determined using a gas chromatograph (PerkinElmer Clarus 580).

The analysis of acetylene reduction activity (ARA) after a shift in high KNO_3_ conditions has been performed as described in the study by D’Apuzzo et al. (2015); nodulated plants were transferred at 4 wpi on slanted Petri dishes where roots are placed in sandwich position between two filter papers wet with a Gamborg B5 liquid media containing no KNO_3_ or 10 mM KNO_3_. Plants are maintained for 48 h in these conditions with filter papers wet with 20 ml liquid media. ARA was then tested as described above.

### Estimation of Anthocyanin

Stem tissue from three plants per assay was weighed and then extracted with 99:1 methanol: HCl (v/v) at 4°C. The OD_530_ and OD_657_ for each sample were measured, and relative anthocyanin levels were determined with the equation OD_530_ – (0.25 × OD_657_) × extraction volume (ml) × 1/weight of tissue sample (g) = relative units of anthocyanin/g fresh weight of tissue.

### Quantitative Real-Time PCR

RNA was extracted as described in Omrane et al. (2009). Real-time PCR was performed with a DNA Engine Opticon 2 System, MJ Research (MA, United States) using SYBR to monitor dsDNA synthesis. The procedure was described in the study by Moscatiello et al. (2018). The ubiquitin (*UBI*) gene (AW719589) was used as an internal standard. The concentration of primers was optimized for every PCR, and amplifications were carried out in triplicate. The PCR program used was as follows: 95°C for 3 min and 39 cycles of 94°C for 15 s, 60°C for 15 s, and 72°C for 15 s. Data were analyzed using Opticon Monitor Analysis Software version 2.01 (MJ Research). The quantitative real-time PCR (qRT-PCR) data were analyzed using the comparative Ct method. The relative level of expression was calculated with the following formula: The relative expression ratio of the gene of interest is 2^−∆CT^ with ∆←←←CT = Ct_GENE_ minus CT_UBI_. The efficiency of the *LjNPF3.1* primers was assumed to be two. Analysis of the melting curve of PCR product at the end of the PCR run revealed a single narrow peak for each amplification product, and fragments amplified from total complementary DNA (cDNA) were gel-purified and sequenced to assure accuracy and specificity. The oligonucleotides used for the qRT-PCR are listed in Supplementary Table S1.

### Plasmid Preparation

The various constructs used for *L. japonicus* transformation were assembled using Golden Gate Cloning (Weber et al., 2011). The *LjNPF3.1* sequences have been retrieved in the Lotus database (Gifu assembly; Mun et al., 2016)^3^ and synthesized by ThermoFisher Scientific. Overhangs extremities with the *Bsa*I or *Bpi*I restriction sites have been added to the sequences. For promoter activity studies, the putative promoter region of 2,503 bp upstream of the translation start of LjNPF3.1 was used to drive a triple YFP reporter carrying a nuclear localization signal on the C terminus (Reid et al., 2016). The construct was assembled in a pIV10-L2 vector backbone (ampicillin and spectinomycin/streptomycin resistant). For localization studies in tobacco protoplasts, the *LjNPF3.1* CDS sequence was cloned downstream of the 35S promoter and upstream of the sequence encoding the eYFP directly into pICH binary vector backbone (Weber et al., 2011). Restriction ligase reactions were made using the *Bpi*I restriction enzyme and T4 ligase of ThermoFisher Scientific.

### Statistical Analysis

Statistical analyses were performed using the VassarStats analysis of variance program.

### Confocal Imaging

For promoter activity using tYFPnls, transformed roots were fixed with paraformaldehyde and cleared as described previously (Warner et al., 2014). The samples were analyzed on a ZEISS confocal microscope LSM780. The whole root images were obtained using Z-stack and tile scan tools with 514/515–530 nm excitation/emission settings. Final images were generated by Maximum Intensity Projection in ZEN software (ZEISS) or ImageJ. For the 35S driven LjNPF3.1-YFP fusion in tobacco protoplast, confocal analyses were performed using a LeicaDMi8 (Leica Biosystems, Wetzlar, Germany) laser scanning confocal imaging system as described in Rogato et al. (2008). For YFP detection, excitation was set at 488 nm, and detection between 515 and 530 nm.

## Results

### *LjNPF3.1* Expression Is Strongly Induced in N_2_-Fixing Nodules

**Figure 1:**
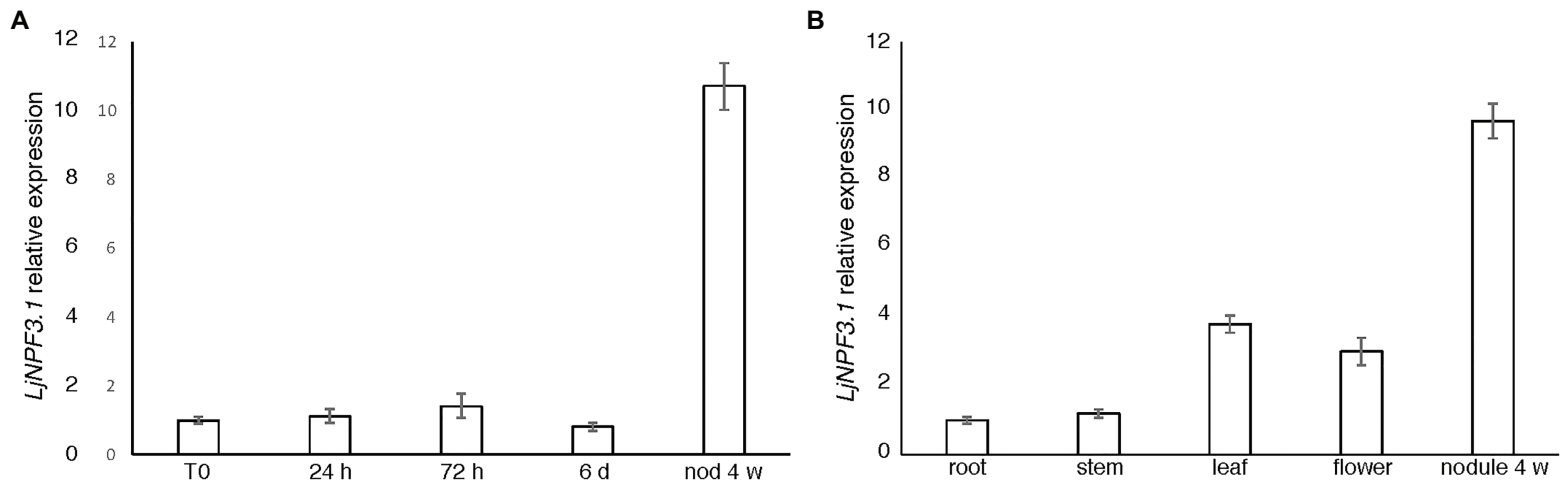
*LjNPF3.1* transcriptional regulation. **(A)** Time-course analysis in wild-type *Lotus japonicus* root and nodule tissues after *Mesorhizobium loti* inoculation. RNAs were extracted from roots of seedlings grown in N-starvation conditions at different times after inoculation (R0, 24 h, 72 h, 6 days, and 14 days) and from mature nodules (28 dpi). **(B)** Expression in different organs. RNAs were extracted 4 weeks after inoculation. Mature flowers were obtained from lotus plants propagated in the growth chamber. Expression levels obtained by quantitative real-time PCR (qRT-PCR) were normalized with respect to the internal control ubiquitin (*UBI*) gene and plotted as relative to the expression of T0 **(A)** and root **(B)**. Data bars represent the mean and SDs of data obtained with RNA extracted from three different sets of plants and three real-time PCR experiments.

We have recently reported the identification of the 86 members of the *L. japonicus* NPF family (accession MG20; Criscuolo et al., 2012; Sol et al., 2019) whose nomenclature was assigned based on the two-letter code established in Léran et al. (2014). The name *LjNPF3.1* is assigned to the MG20 gene *Lj2g3v1155500.1* (Sol et al., 2019) and the identical copy *LotjaGi2g1v0278100.1*, identified in the *L. japonicus* accession Gifu (Supplementary Table S2; Kamal et al., 2020).^4^ *LjNPF3.1* is one of the three members of the clade 3 (Supplementary Table S2) coding for a 580-amino acid protein with a molecular mass of 64.4 kDa. LjNPF3.1 has 12 TM predicted domains (Tusnàdy and Simon, 2001) and shares the highest level of amino acid identity among the *Arabidopsis* NPF members, with the AtNPF3.1 protein (74%; AT1G68570.1). *LjNPF3.1* was previously included in the subclass of eight *LjNPF* nodule-induced genes identified by *in silico* analysis (Valkov and Chiurazzi, 2014).^5^ We have now confirmed this specific profile of expression in a time-course experiment where *L. japonicus* seedlings grown in a derived Gamborg-B5 medium without N sources were inoculated with *M. loti* 1 week after sowing. The *LjNPF3.1* transcript was barely detectable in uninoculated as well as roots tested up to 14 days after inoculation, whereas a strong induction was observed in mature nodules (tenfold induction; Figure 1A). To further characterize the profile of expression of *LjNPF3.1*, we have also analyzed the distribution of the *LjNPF3.1* transcript in different organs of *L. japonicus*. The *LjNPF3.1* gene showed a regulated transcriptional profile with a peak of expression in mature nodules and a significant level of transcript in leaves and mature flowers (Figure 1B).

### *LjNPF3.1* Is Expressed in Root and Nodule Cortical Regions

**Figure 2:**
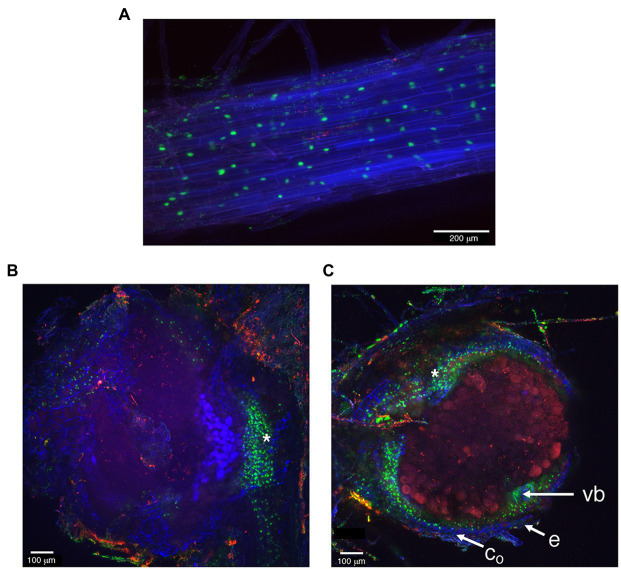
The activity of *LjNPF3.1* 2.5-kb promoter fused to the tYFP-NLS reporter (nuclear-localized, green) in inoculated hairy roots. **(A)** Expression in the root cortical region. **(B)** Increased YFP fluorescence was detected at the base of mature nodules. **(C)** YFP fluorescence at the periphery of the N_2_-fixing nodules in the outer cortex region. *, nodule base; vb, vascular bundle; c_o_, outer cortex; e, epidermis.

To gain information about the spatial distribution of the *LjNPF3.1* transcript in root and nodule tissues, the putative promoter region of the *LjNPF3.1* gene was obtained by amplifying a PCR fragment extending up to 2.5 kb upstream of the ATG. A *proNPF3.1:tYFP-NLS* reporter constructs localizing triple YFP in the nucleus of transgenic roots was used to visualize the *LjNPF3.1* spatial gene expression at the cellular level. Confocal microscopy analyses indicated that the *LjNPF3.1* promoter was active in the cortical cells of the inoculated hairy roots (Figure 2A), and the fluorescence level increased at the base of the nodules (Figure 2B), consistent with the increased expression shown in Figure 1. The section in Figure 2C shows a strong YFP signal also in the peripheral parenchymatic nodule region, most likely the outer cortex cell layers.

**Figure 3:**
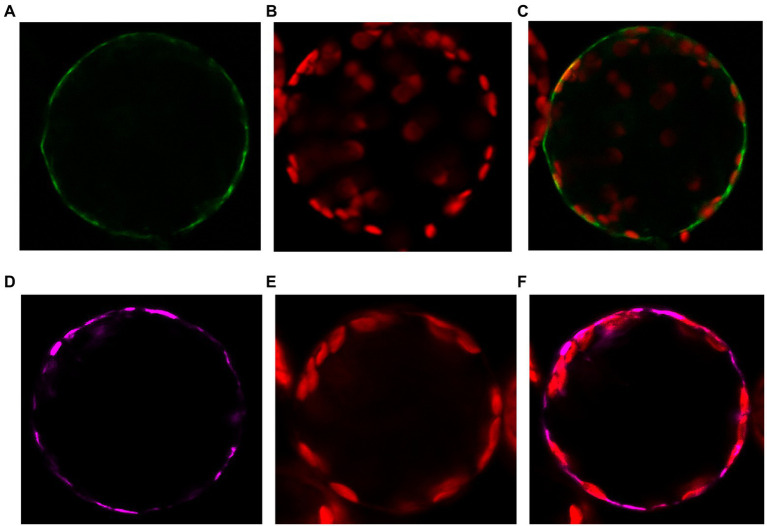
Plasma membrane localization of the p35S-LjNPF3.1-eYFP fusion transiently expressed in protoplasts of tobacco mesophyll cells. **(A)** Plasma membrane localization of the 35S-LjNPF3.1-eYFP. **(B)** Chlorophyll autofluorescence of the transformed protoplast. **(C)** Merged image of **(A,B)**. **(D)** Plasma membrane localization of the pUBI-AtPIP2A-mCherry marker (purple). **(E)** Chlorophyll autofluorescence of the transformed protoplast. **(F)** Merged image of **(C,D)**.

To determine the LjNPF3.1 subcellular localization, we generated a construct that fused YFP to the C-terminus of LjNPF3.1 under the control of the 35S promoter and 35S terminator and introduced this construct in tobacco protoplasts. Although we did not provide evidence that the 35S-LjNPF3.1-YFF fusion maintains the correct biological function, confocal microscopy analysis indicated that this localizes at the plasma membrane of transformed protoplasts, mirroring the localization of the AtPIP2A-mCherry plasma membrane marker (Figures 3A–F).

### Isolation of LORE1-Insertion Null Mutants and Phenotypic Characterization

**Figure 4:**
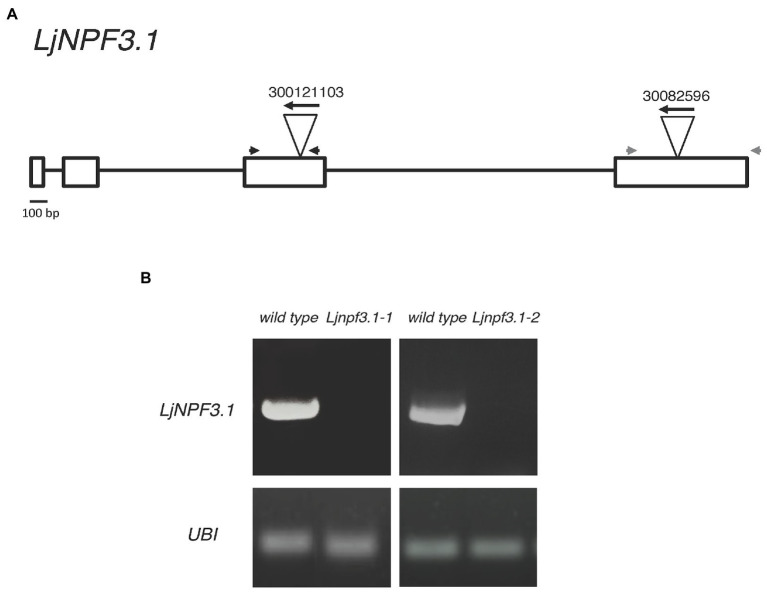
**(A)** Exon/intron organization of the *LjNPF3.1* gene. Insertion sites, relative orientations of the LORE1 retrotransposon element in the 300121103 and 30082596 lines, and positions of a couple of primers (gray and black arrowheads) used for genotyping are indicated. **(B)** *LjNPF3.1* is not expressed in LORE1 homozygous mutant lines.

**Figure 5:**
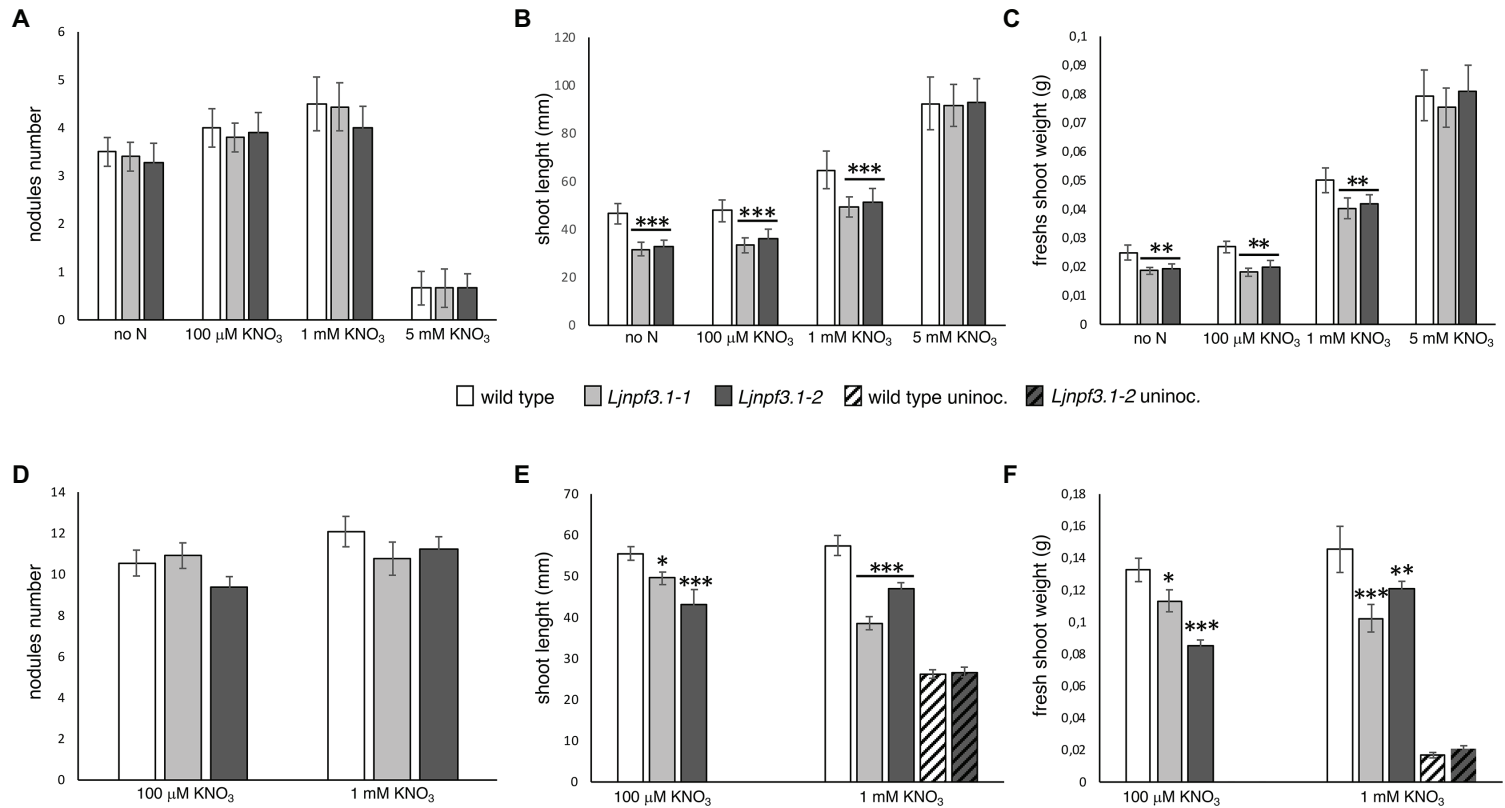
Phenotypic characterization of *Ljnpf3.1-1* and *Ljnpf3.1-2* mutants. Wild-type and *Ljnpf3-1* plants, inoculated with *M. loti* and grown in the presence of different concentrations of KNO_3_. **(A–C)** Plants grown in Petri dishes, axenic conditions, scored 4 weeks after inoculation. **(D–F)** Plants were grown in pots scored at 6 weeks after inoculation. **(A,D)** Nodules number per plant. **(B,D)** Shoots length per plant. **(C,F)** Shoots fresh weight per plant. Different KNO_3_ regimes and plant genotypes are indicated [noninoculated plants in panels **(E,F)** are indicated by oblique-filled bars]. Bars represent the means and SE of measures from three experiments (at least 20 plants per experiment per condition). Asterisks indicate significant differences with wild-type levels. ^∗^*p* < 0.05; ^∗∗^*p* < 0.002; and ^∗∗∗^*p* < 0.0001.

**Figure 6:**
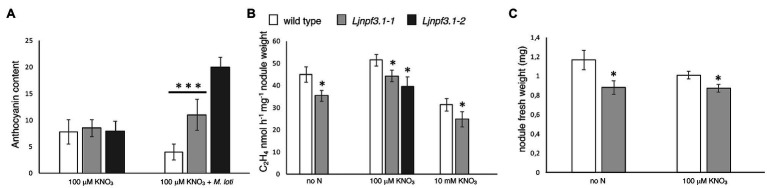
Phenotypic characterization of *Ljnpf3.1-1* and *Ljnpf3.1-2* mutants. **(A)** Analysis of anthocyanin content in the stems of wild-type and *Ljnpf3.1* plants grown in Petri dishes with/without *M. loti* inoculation. Relative anthocyanin levels scored in the *Y*-axis were determined with the equation OD_530_ – (0.25 × OD_657_) × extraction volume (ml) × 1/weight of tissue sample (g) = relative units of anthocyanin/g fresh weight of tissue. Anthocyanin was extracted 4 weeks after sowing. **(B)** Acetylene reduction activity (ARA) per nodule weight at 4 weeks postinoculation. **(C)** Nodules fresh weight. Plants were grown in Petri dishes as in Figures 5A–C. N regimes and plant genotypes are indicated. Data bars represent the means and SE of measures from six experiments in (**B**; eight samples of five nodules per experiment per condition). Asterisks indicate significant differences with wild-type levels. ^∗^*p* < 0.05 and ^∗∗∗^*p* < 0.001.

To determine the *in vivo* function of *LjNPF3.1*, two independent LORE1 insertion mutants have been isolated from the LORE1 lines collection established in the *L. japonicus* Gifu accession (Fukai et al., 2012; Urbanski et al., 2012; Malolepszy et al., 2016). Lines 300121103 and 30082596, bearing retrotransposon insertions in the third and fourth exon (Figure 4A), have been genotyped by PCR, and plants homozygous for those insertion events were selected and transferred to the plant chamber for the production of seeds. Endpoint RT-PCR analyses of homozygous plants from lines 300121103 and 30082596 revealed no detectable *LjNPF3.1* mRNA in mature nodules and hence, considered null mutants and hereafter named *Ljnpf3.1-1* and *Ljnpf3.1-2*, respectively (Figure 4B). Two individual homozygous mutant plants from each insertion line have been selected for analyses, and because their growth phenotypes did not significantly differ, the data obtained with the selected individual mutants have been pooled. As transcript abundance and promoter activity were related to mature nodules, we analyzed the symbiotic performances of the *Ljnpf3.1* plants in terms of nodule formation capacity and shoot growth parameters exhibited at 4 weeks after inoculation with *M. loti*. Wild-type and *Ljnpf3.1* synchronized seedlings with a 0.5 cm long root were transferred 5 days after sowing, in Petri dishes containing B5-derived medium without N sources or with different concentrations of KNO_3_ and inoculated with *M. loti*. Wild-type and mutant plants display the same nodule formation capacity, whereas a clear-cut reduction of the shoot length (21–32%) and fresh weight (16–26%) values were exhibited by *Ljnpf3.1-1* and *-2* when compared to the ones of the wild-type plants (Figures 5A–C; Supplementary Figure S1). Importantly, when the growth medium was supplemented with 5 mM KNO_3_, a concentration that is known to inhibit nodule initiation (Barbulova et al., 2007) and sufficient to support optimal plant growth, the mutants did not display any shoot growth-defective phenotype (Figures 5A–C; Supplementary Figure S1). The specificity of the symbiotic deficient phenotype was further confirmed by another set of experiments where wild-type and mutant genotypes were grown in pots to allow the evaluation of symbiotic phenotypes up to 6 weeks after inoculation. The scoring of nodulation, as well as shoot growth parameters, confirmed the deficient phenotype of both *Ljnpf3.1* inoculated plants, whereas no differences were observed in uninoculated plants grown in the presence of 1 mM KNO_3_ (Figures 5D–F; Supplementary Figure S2). Furthermore, as a correlation between nitrate uptake/reallocation/assimilation and water stress responses has been well-characterized in plants (Chen et al., 2012; Gloser et al., 2020), we have also analyzed the dry weight of detached wild-type and mutant leaves to rule out the hypothesis that the reduced weight was due to different water contents. Data shown in Supplementary Figure S3 indicate that the dry weight of leaves was also significantly reduced in mutants as compared to wild-type and that the wt/*Ljnpf3.1* fresh and dry weight ratios remain constant. The specific symbiotic deficient phenotype displayed by the *Ljnpf3.1* plants was also confirmed by the substantially increased accumulation of anthocyanin exhibited in the stems of the inoculated mutants as compared to wild-type plants, with the exception of plants grown in the presence of sufficient N conditions (5 mM KNO_3_; Supplementary Figure S1). The anthocyanin accumulation was quantified, and the data shown in Figure 6 indicate a 3–5-fold increase in the mutants as compared to wild-type, whereas such a difference was not scored in the uninoculated plants (Figure 6A).

To test whether the symbiotic phenotypes could be associated with altered nodule functionality, we have analyzed the N_2_-fixing performances of wild-type and *Ljnpf3.1* mutant nodules. The ARA was measured in detached nodule samples from five independent experiments in plants grown in axenic conditions at 4 weeks postinoculation. A significant 22% reduction of activity in plants grown without N supply was scored, and this deficiency was reduced to still a significant 14% (*Ljnpf3.1-1*) and 24% (*Ljnpf3.1-2*) in nodules of mutants grown in the presence of 100 μM KNO_3_ (Figure 6B). Furthermore, the comparison of nodulation phenotypes in nitrate-free and 100 μM KNO_3_ revealed a significant increase in nodule biomass (fresh weight) in the wild-type as compared to *Ljnpf3.1-1* (Figure 6C; 25 and 15%, respectively). To test whether *LjNPF3.1* is involved in the pathway responsible for the rapid drop of the N_2_-fixation activity observed in nodules after the transfer in the presence of high external nitrate concentrations (Arrese-Igor et al., 1998; Cabeza et al., 2014), we compared the values of acetylene reduction in wild-type and mutant nodules after transfer in the presence of 10 mM KNO_3_ for 48 h. As shown in Figure 6B, the N_2_-fixation capacity is reduced to more than 30% in both wild-type and mutant nodules after the transfer in high nitrate conditions (Figure 6B).

The identical phenotypes displayed by the *Ljnpf3.1-1* and *-2* null mutants confirmed that the LORE1 insertions in the *LjNPF3.1* gene are the causal mutations of the deficient phenotypes observed exclusively in symbiotic conditions. In addition, heterozygous plants for the LORE1 insertion, the *LjNPF3.1* gene isolated in the two analyzed lines, displayed neither the shoot biomass deficient phenotypes nor the anthocyanin accumulation (data not shown).

## Discussion

The important contribution of transporters of the NPF family to the functioning of N_2_-fixing nodules is suggested by the large numbers of *NPF* genes found to be upregulated in mature nodules (Takanashi et al., 2012; Valkov and Chiurazzi, 2014; Clarke et al., 2015; Wang et al., 2016). In this study, we report the characterization of one of the eight upregulated *LjNPF* genes identified in mature N_2_-fixing *Lotus* nodules, *LjNPF3.1* (Valkov and Chiurazzi, 2014). Analyses of the expression profile revealed an increase in the *LjNPF3.1* transcript in nodules compared to roots (more than 10-fold; Figure 1). The clade 3 of the *L. japonicus* NPF family consists of three members as in other legume plants (*Medicago truncatula* and *Phaseolus vulgaris*; Léran et al., 2014), and *LjNPF3.1* is the only one upregulated in mature nodules.^6^ Interestingly, *MtNPF3.1* that shares 84% of amino acid identity with *LjNPF3.1* also exhibits the induced profile of expression in mature nodules (He et al., 2009).^7^

**Figure 7:**
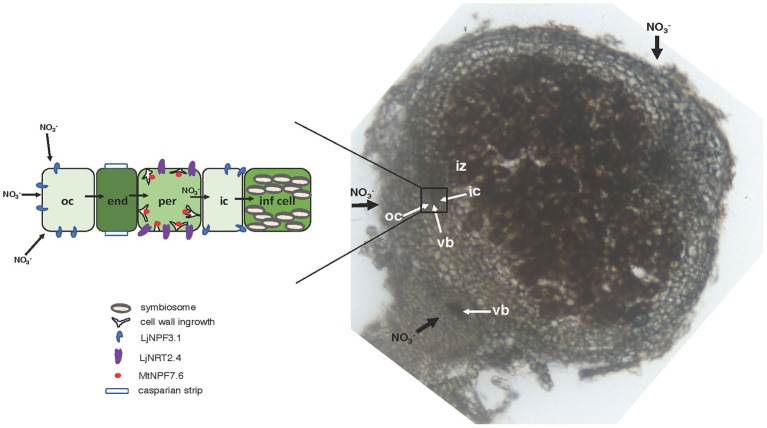
Model of nitrate flux in the N_2_-fixing nodule. Right side: 70 μm vibratome longitudinal section of a 4-week old *L. japonicus* nodule with an illustration of the flux of nitrate (thick black arrows) toward the infected cells of the N_2_-fixation zone either from the root or from outside to inside the nodule. The rectangle indicates the vascular bundle structure bracketed by the outer and inner cortex. Left side: scheme of the flux of nitrate through different cell layers with an illustration of the NPFs and NRT2 involved. The NPFs and NRT2 transporters involved in the transport of nitrate, subcellular structures, and organelles are indicated. oc, outer cortex; ic, inner cortex; vb, vascular bundle; iz, infection zone; end, endodermis; per, pericycle; inf cell, infected cell.

The phenotypic characterization of the two independent null mutants provides evidence for a positive role played by *LjNPF3.1* for an efficient nodule functioning (Figures 5, 6). The reduction of shoot height and weight scored in the mutants in the whole range of permissive low N conditions tested (Figures 5B,C,E,F) might be explained, in the presence of an equal number of nodules (Figures 5A,D), by the partial, but significant, impairment of the nitrogenase activity reported in Figure 6B. Consistently, the shoot phenotypes displayed by the *Ljnpf3.1* mutants (Figure 5; Supplementary Figure S1) are not as severe as those reported for the *fix^−^* mutants, rather resembling the ones of the mutants described as Fix^+^/Fix^−^, which show an impaired N_2_-fixation activity (Garcìa-Calderon et al., 2012; Pislariu et al., 2012). The relatively slight reduction of nitrogenase activity could also be due to the functional redundancy of *LjNPF3.1* with other *NPF* genes upregulated in the mature nodule (Valkov and Chiurazzi, 2014). The relationship between the reduced shoot biomass and the N-starvation condition due to the partial efficiency of the N_2_-fixation activity is confirmed by the rescue of the stressed phenotype observed in the presence of 5 mM KNO_3_ (Figures 5B,C). Furthermore, the anthocyanin accumulation scored only in the stems of the inoculated *Ljnpf3.1* plants represents another indication of an impaired N_2_-fixation activity in the mutant genetic background as anthocyanin accumulation in the stems has been reported as a phenotype associated with N-starvation condition associated with a deficiency of the nodule functioning or lack of nodulation (Krussell et al., 2005; Ott et al., 2005; Bourcy et al., 2013; Pal’ove-Balang et al., 2015). Although we cannot exclude the possibility that the spatial expression of *LjNPF3.1* is also controlled by sequences located outside the 2.5 kb of the 5'-UTR region used in the promoter-fusion construct, the spatial expression profile (Figure 2) provides some clues about the action played by *LjNPF3.1* for the control of nodule efficiency. The preferential expression in the root cortex shown in Figure 2A has been only reported for two *Arabidopsis* nitrate excretion transporters (NAXT) of the subgroup 2, AtNPF2.5 and AtNPF2.7, which mediate chloride and nitrate efflux from the root, respectively (Segonzac et al., 2007; Li et al., 2017). Recently, in *M. truncatula*, the flux of nitrate from the root tissue and from the external environment to the nodules has been traced (Wang et al., 2020). The MtNPF7.6 high-affinity nitrate transporter, specifically expressed in nodule vascular tissues, functions in nitrate uptake and transport through the nodule transfer cells (NTC) to fine-tune the nodule development and functioning in response to fluctuating environmental nitrate status (Wang et al., 2020). The spatial expression profile of *LjNPF3.1* in the root cortical region at the base of the nodule (Figure 2B) and in the layers of cortex adjacent to the infected cells inside the nodule (Figure 2C) is consistent with possible involvement in the flux of nitrate from the root and from outside to inside the nodule toward the N_2_-fixation zone (Figure 7) where nitrate might contribute to regulate NO homeostasis and oxidative stress (Horchani et al., 2011; Hicri et al., 2015; Valkov et al., 2017, 2020; Signorelli et al., 2020; Villar et al., 2020; Wang et al., 2020). However, the involvement of *LjNPF3.1* should be limited to conditions of low external concentration that are permissive for nodule functioning, as the inhibitory pathway occurring at high concentrations of nitrate is not altered in the *Ljnpf3.1* mutants (Figure 6B). So far, it is noteworthy that the transporters reported being involved in the nodular transport of nitrate (Figure 7) exhibit capacity to transport this ion at low concentrations (0.5 mM; Valkov et al., 2017), with saturable kinetics in the micromolar range (Wang et al., 2020) or as members of the high-affinity NRT2 family (Valkov et al., 2020).

In *A. thaliana*, the clade 3 of the NPF family consists of a unique member, AtNPF3.1. AtNPF3.1 has also been reported to act as a low-affinity nitrate/nitrite transporter in *Xenopus laevis* oocytes (Pike et al., 2014) although the nitrate transport capacity could not be confirmed by the characterization of *Atnpf3.1* mutants (David et al., 2016). However, as already mentioned, it has been well-documented that NPFs transport various substrates, including phytohormones, and hence, the involvement of *LjNPF3.1* in other pathways such as the one governing the distribution of gibberellin (GA), recently reported as a positive regulator of nodule functioning (Serova et al., 2019), cannot be excluded.

In conclusion, although a further characterization of the *LjNPF3.1* gene will certainly pass through the identification of the preferentially transported substrate(s), the data reported here clearly indicate its crucial role in the correct N_2_-fixation process, further highlighting the roles played by NPF transporters for an efficient nodule functioning.

## Data Availability Statement

The datasets presented in this study can be found in online repositories. The names of the repository/repositories and accession number(s) can be found in the article/**Supplementary Material**.

## Author Contributions

YV, MN, and AR designed, performed, and analyzed the experiments. SR designed and analyzed the experiments. VV designed, performed, and analyzed the experiments and contributed to the writing of this manuscript. MC designed the experiments and wrote the article. YV, MN, AR, SR, VV, and MC contributed to the experimental design and manuscript preparation. All authors contributed to the article and approved the submitted version.

### Conflict of Interest

The authors declare that the research was conducted in the absence of any commercial or financial relationships that could be construed as a potential conflict of interest.

## Footnotes

^1^http://www.weber.dk

^2^http://www.damolin.dk

^3^https://lotus.au.dk/

^4^https://lotus.au.dk/

^5^https://lotus.au.dk/expat/

^6^https://lotus.au.dk/expat/

^7^https://mtgea.noble.org/v2/

## Supplementary Material

Supplementary Figure S1 Representative images of the wild-type and *Ljnpf3.1* plants at 4 weeks after inoculation. Nitrate conditions and plant genotypes are indicated. The white arrows indicate the zones displaying the evident accumulation of anthocyanin in the stems of the mutant plants as compared to the wild-type.

Supplementary Figure S2 Representative images of the wild-type and *Ljnpf3.1* plants at 6 weeks after inoculation. Nitrate condition and plant genotypes are indicated. The white arrows are pointed on stems of mutants displaying evident accumulation of anthocyanin.

Supplementary Figure S3 Phenotypic characterization of *Ljnpf3.1-1* and *Ljnpf3.1-2* mutants. **(A)** Fresh weight of detached leaves. **(B)** The dry weight of detached leaves. Plants were grown in Petri dishes, in the presence of 1 mM KNO_3_. Leaves from single plants were detached and immediately weighted at 4 weeks after inoculation. Bars represent the means and SE of measures from three experiments (12 plants per experiment). Asterisks indicate significant differences with wild-type levels. ^*^*p* < 0.05.

Supplementary Table S1 Oligonucleotides are used in the present work.

Supplementary Table S2 Description of the *Lotus japonicus* NPF clade 3.

## References

[ref1] Appleby C. A. (1984). Leghemoglobin and *Rhizobium* respiration. Annu. Rev. Plant Physiol. Plant Mol. Biol. 35, 443–478. 10.1146/annurev.pp.35.060184.002303

[ref2] Arrese-Igor C. Gordon A. J. Minchin F. R. Denison R. F. (1998). Nitrate entry and nitrite formation in the infected region of soybean nodules. J. Exp. Bot. 49, 41–48. 10.1093/jxb/49.318.41

[ref3] Barbulova A. D’Apuzzo E. Rogato A. Chiurazzi M. (2005). Improved procedures for in vitro regeneration and for phenotypical analysis in the model legume *Lotus japonicus*. Funct. Plant Biol. 32, 529–536. 10.1071/FP05015, PMID: 32689153

[ref4] Barbulova A. Rogato A. D’Apuzzo E. Omrane S. Chiurazzi M. (2007). Differential effects of combined N sources on early steps of the Nod factor-dependent transduction pathway in *Lotus japonicus*. Mol. Plant-Microbe Interact. 20, 994–1003. 10.1094/MPMI-20-8-0994, PMID: 17722702

[ref5] Bastianelli F. Costa A. Vescovi M. D’Apuzzo E. Zottini M. Chiurazzi M.. (2009). Salicylic acid differentially affects suspension cell cultures of *Lotus japonicus* and one of its non-symbiotic mutants. Plant Mol. Biol. 72, 469–483. 10.1007/s11103-009-9585-8, PMID: 20012170

[ref6] Bergensen F. J. (1996). Delivery of O_2_ to bacteroids in soybean nodule cells: considerations of gradients of concentrations of free, dissolved O_2_ in and near symbiosomes and beneath intercellular spaces. Protoplasma 191, 9–20. 10.1007/BF01280821

[ref7] Bourcy M. Brocard L. Pislariu C. I. Cosson V. Mergaert P. Tadege M.. (2013). *Medicago truncatula* DNF2 is a PI-PLC-XD-containing protein required for bacteroid persistence and prevention of nodule early senescence and defence-like reactions. New Phytol. 197, 1250–1261. 10.1111/nph.12091, PMID: 23278348

[ref8] Cabeza R. Koester B. Liese R. Lingner A. Baumgarten V. Dirks J.. (2014). An RNA sequencing transcriptome analysis reveals novel insights into molecular aspects of the nitrate impact on the nodule activity of *Medicago truncatula*. Plant Physiol. 164, 400–411. 10.1104/pp.113.228312, PMID: 24285852 PMC3875817

[ref9] Carroll B. Gresshoff P. M. (1983). Nitrate inhibition of nodulation and nitrogen fixation in white clover. Z. Pflanzenphysiol. 110, 77–88. 10.1016/S0044-328X(83)80218-9

[ref10] Chen C. Z. Lv X. F. Li J. Y. Yi H. Y. Gong J. M. (2012). *Arabidopsis* NRT1.5 is another essential component in the regulation of nitrate reallocation and stress tolerance. Plant Physiol. 159, 1582–1590. 10.1104/pp.112.199257, PMID: 22685171 PMC3425198

[ref11] Clarke V. C. Loughlin C. Gavrin A. Chen C. Brear E. M. Day D. A.. (2015). Proteomic analysis of the soybean symbiosome identifies new symbiotic proteins. Mol. Cell. Proteomics 14, 1301–1322. 10.1074/mcp.M114.043166, PMID: 25724908 PMC4424401

[ref12] Corratgé-Faillie C. Lacombe B. (2017). Substrate (un)specificity of *Arabidopsis* NRT1/PTR family (NPF) proteins. J. Exp. Bot. 68, 3107–3113. 10.1093/jxb/erw499, PMID: 28186545

[ref13] Criscuolo G. Valkov V. T. Parlati A. Martin-Alves L. Chiurazzi M. (2012). Molecular characterization of the *Lotus japonicus* NRT1 (PTR) and NRT2 families. Plant Cell Environ. 35, 1567–1581. 10.1111/j.1365-3040.2012.02510.x, PMID: 22458810

[ref14] D’Apuzzo E. Valkov V. T. Parlati A. Omrane S. Barbulova A. Sainz M. M.. (2015). PII overexpression in *Lotus japonicus* affects nodule activity in permissive low nitrogen conditions and increases nodule numbers in high nitrogen treated plants. Mol. Plant-Microbe Interact. 28, 432–442. 10.1094/MPMI-09-14-0285-R, PMID: 25390190

[ref15] David L. C. Berquin P. Kanno Y. Seo M. Daniel-Vedele A. Ferrario-Mery S. (2016). N availability modulates the role of NPF3.1, a gibberellin transporter, in GA-mediated phenotypes in *Arabidopsis*. Planta 244, 1315–1328. 10.1007/s00425-016-2588-1, PMID: 27541496

[ref16] Frommer W. B. Hummel S. Rentsch D. (1994). Cloning of an Arabidopsis histidine transporting protein related to nitrate and peptide transporters. FEBS Lett. 347, 185–189. 10.1016/0014-5793(94)00533-8, PMID: 8033999

[ref17] Fujikake H. Yamazaki A. Ohtake N. Sueyoshi K. Matsuhashi S. Ito T.. (2003). Quick and reversible inhibition of soybean nodule growth by nitrate involves a decrease in sucrose supply to nodules. J. Exp. Bot. 54, 1379–1388. 10.1093/jxb/erg147, PMID: 12709484

[ref18] Fukai E. Soyano T. Umehara Y. Nakayama S. Hirakawa H. Tabata S.. (2012). Establishment of a *Lotus japonicus* gene tagging population using the exon-targeting endogenous retrotransposon LORE1. Plant J. 69, 720–730. 10.1111/j.1365-313X.2011.04826.x, PMID: 22014259

[ref19] Gamborg O. L. (1970). The effects of amino acids and ammonium on the growth of plant cells in suspension culture. Plant Physiol. 45, 372–375. 10.1104/pp.45.4.372, PMID: 16657321 PMC396416

[ref20] Garcìa-Calderon M. Chiurazzi M. Espuny M. R. Màrquez A. J. (2012). Photorespiratory metabolism and nodule function. Behavior of *Lotus japonicus* mutants deficient in plastid glutamine synthetase. Mol. Plant-Microbe Interact. 25, 211–219. 10.1094/MPMI-07-11-0200, PMID: 22007601

[ref21] Gloser V. Dvoraskova M. Mota D. H. Petrovic B. Gonzalez P. Geilfus C. M. (2020). Early changes in nitrate uptake and assimilation under drought in relation to transpiration. Front. Plant Sci. 11:602065. 10.3389/fpls.2020.602065, PMID: 33424901 PMC7793686

[ref22] Handberg K. Stougaard J. (1992). *Lotus japonicus*, an autogamous, diploid legume species for classical and molecular genetics. Plant J. 2, 487–496. 10.1111/j.1365-313X.1992.00487.x

[ref23] He J. Benedito V. A. Wang M. Murray J. D. Zhao P. X. Tang Y.. (2009). The *Medicago truncatula* gene expression atlas web server. BMC Bioinformatics 10:441. 10.1186/1471-2105-10-441, PMID: 20028527 PMC2804685

[ref24] Hicri I. Boscari A. Castella C. Rovere M. Puppo A. Brouquisse R. (2015). Nitric oxide: a multifaceted regulator of the nitrogen-fixing symbiosis. J. Exp. Bot. 66, 2877–2887. 10.1093/jxb/erv051, PMID: 25732535

[ref25] Horchani F. Prévot M. Boscari A. Evangelisti E. Meilhoc E. Bruand C.. (2011). Both plant and bacterial nitrate reductases contribute to nitric oxide production in *Medicago truncatula* nitrogen-fixing nodules. Plant Physiol. 155, 1023–1036. 10.1104/pp.110.166140, PMID: 21139086 PMC3032450

[ref26] Jeong J. Suh S. Guan C. Tsay Y. F. Moran N. Oh C. J.. (2004). A nodule-specific dicarboxylate transporter from alder is a member of the peptide transporter family. Plant Physiol. 134, 969–978. 10.1104/pp.103.032102, PMID: 15001700 PMC389920

[ref27] Jiang Q. Gresshoff P. M. (1997). Classical and molecular genetics of the model legume *Lotus japonicus*. Mol. Plant-Microbe Interact. 10, 59–68. 10.1094/MPMI.1997.10.1.59, PMID: 9002271

[ref76] Kamal N. Man T. Reid D. Lin J. S. Akyol T. Y. Sandal N.. (2020). Insights into the evolution of symbiosis gene copy number and distribution from a chromosome-scale Lotus japonicus Gifu genome sequence. DNA Res. 1:27. 10.1093/dnares/dsaa015 PMC7508351 32658273

[ref28] Kanno Y. Hanada A. Chiba Y. Ichikawa T. Nakazawa M. Matsui M.. (2012). Identification of an abscisic acid transporter by functional screening using the receptor complex as a sensor. Proc. Natl. Acad. Sci. U. S. A. 109, 9653–9658. 10.1073/pnas.1203567109, PMID: 22645333 PMC3386071

[ref29] Krouk G. Lacombe B. Bielach A. Perrine-Walker F. Malinska K. Mounier E.. (2010). Nitrate-regulated auxin transport by NRT1.1 defines a mechanism for nutrient sensing in plants. Dev. Cell 18, 927–397. 10.1016/j.devcel.2010.05.008, PMID: 20627075

[ref30] Krussell L. Krause K. Ott T. Desbrosses G. Kramer U. Sato S.. (2005). The sulfate transporter SST1 is crucial for symbiotic nitrogen fixation in *Lotus japonicus* root nodules. Plant Cell 17, 1625–1636. 10.1105/tpc.104.030106, PMID: 15805486 PMC1091779

[ref31] Léran S. Varala K. Boyer J. C. Chiurazzi M. Crawford N. Daniel-Vedele F.. (2014). A unified nomenclature of nitrate transporter 1/peptide transporter family members in plants. Trends Plant Sci. 19, 5–9. 10.1016/j.tplants.2013.08.008, PMID: 24055139

[ref32] Li B. Qiu J. Jayakannan M. Xu B. Li Y. Mayo G. M.. (2017). *AtNPF2.5* modulates chloride (Cl^−^) efflux from roots of *Arabidopsis thaliana*. Front. Plant Sci. 7:2013. 10.3389/fpls.2016.02013, PMID: 28111585 PMC5216686

[ref33] Liu K. H. Huang C. Y. Tsay Y. (1999). CHL1 is a dual-affinity nitrate transporter of *Arabidopsis* involved in multiple phases of nitrate uptake. Plant Cell 11, 865–874. 10.1105/tpc.11.5.865, PMID: 10330471 PMC144217

[ref34] Liu K. H. Tsay Y. F. (2003). Switching between the two action modes of the dual-affinity nitrate transporter CHL1 by phosphorylation. EMBO J. 22, 1005–1013. 10.1093/emboj/cdg118, PMID: 12606566 PMC150351

[ref35] Malolepszy A. Mun T. Sandal N. Gupta V. Dubin M. Urbański D. F.. (2016). The LORE1 insertion mutant resource. Plant J. 88, 306–317. 10.1111/tpj.13243, PMID: 27322352

[ref36] Miller A. J. Cramer M. D. (2005). Root nitrogen acquisition and assimilation. Plant Soil 274, 1–36. 10.1007/s11104-004-0965-1

[ref37] Moscatiello R. Sello S. Ruocco M. Barbulova A. Cortese E. Nigris S.. (2018). The hydrophobin HYTLO1 secreted by the biocontrol fungus *Trichoderma longibrachiatum* triggers a NAADP-mediated calcium signalling pathway in *Lotus japonicus*. Int. J. Mol. Sci. 19, 2596–2612. 10.3390/ijms19092596, PMID: 30200468 PMC6164116

[ref38] Mun T. Bachmann A. Gupta V. Stougaard J. Andersen S. U. (2016). Lotus base: an integrated information portal for the model legume *Lotus japonicus*. Sci. Rep. 6:39447. 10.1038/srep39447, PMID: 28008948 PMC5180183

[ref39] Naudin C. Corre-Hellou G. Voisin A. S. Oury V. Salon C. Crozat Y.. (2011). Inhibition and recovery of symbiotic N_2_ fixation by peas (*Pisum sativum* L.) in response to short-term nitrate exposure. Plant Soil 346, 275–287. 10.1007/s11104-011-0817-8

[ref40] Nour-Eldin H. H. Andersen T. G. Burow M. Madsen S. R. Jørgensen M. E. Olsen C. E.. (2012). NRT/PTR transporters are essential for translocation of glucosinolate defence compounds to seeds. Nature 488, 531–534. 10.1038/nature11285, PMID: 22864417

[ref41] Omrane S. Chiurazzi M. (2009). A variety of regulatory mechanisms are involved in the nitrogen-dependent modulation of the nodule organogenesis program in legume roots. Plant Signal. Behav. 4, 1066–1068. 10.4161/psb.4.11.9735, PMID: 20009551 PMC2819515

[ref42] Omrane S. Ferrarini A. D’Apuzzo E. Rogato A. Delledonne M. Chiurazzi M. (2009). Symbiotic competence in *Lotus japonicus* is affected by plant nitrogen status: transcriptomic identification of genes affected by a new signalling pathway. New Phytol. 183, 380–394. 10.1111/j.1469-8137.2009.02873.x, PMID: 19500268

[ref43] Ott T. van Dongen J. T. Gunther C. Krussell L. Desbrosses G. Vigeolas H.. (2005). Symbiotic leghemoglobins are crucial for nitrogen fixation in legume root nodules but not for general plant growth and development. Curr. Biol. 15, 531–535. 10.1016/j.cub.2005.01.042, PMID: 15797021

[ref44] Pal’ove-Balang P. Garcia-Calderòn M. Perez-Delgado C. M. Pavlokin J. Màrquez A. J. (2015). A *Lotus japonicus* mutant defective in nitrate uptake is also affected in the nitrate response to nodulation. Plant Biol. 17, 16–25. 10.1111/plb.12169, PMID: 24673996

[ref45] Parker J. L. Newstead S. (2014). Molecular basis of nitrate uptake by the plant nitrate transporter NRT1.1. Nature 507, 68–72. 10.1038/nature13116, PMID: 24572366 PMC3982047

[ref46] Pedrazzini E. Giovinazzo G. Bielli A. de Virgilio M. Frigerio L. Pesca M.. (1997). Protein quality control along the route to the plant vacuole. Plant Cell 9, 1869–1880. 10.1105/tpc.9.10.1869, PMID: 9368420 PMC157028

[ref47] Pike S. Gao F. Kim S. H. Schachtman D. P. Gassmann W. (2014). Members of the NPF3 transporter subfamily encode pathogen-inducible nitrate/nitrite transporters in gravepine and *Arabidopsis*. Plant Cell Physiol. 55, 162–170. 10.1093/pcp/pct167, PMID: 24259683

[ref48] Pislariu C. I. Murray J. D. Wen J. Q. Cosson V. Muni R. R. D. Wang M.. (2012). A *Medicago truncatula* tobacco retrotransposon insertion mutant collection with defects in nodule development and symbiotic nitrogen fixation. Plant Physiol. 159, 1686–1699. 10.1104/pp.112.197061, PMID: 22679222 PMC3425206

[ref49] Reid D. E. Heckmann A. B. Novák O. Stougaard J. (2016). Cytokinin oxidase/dehydrogenase 3 maintains cytokinin homeostasis during root and nodule development in *Lotus japonicus*. Plant Physiol. 170, 1060–1074. 10.1104/pp.15.00650, PMID: 26644503 PMC4734552

[ref50] Rogato A. D’Apuzzo E. Barbulova A. Omrane S. Stedel C. Simon-Rosin U.. (2008). Tissue-specific down-regulation of LjAMT1;1 compromises nodule function and enhances nodulation in *Lotus japonicus*. Plant Mol. Biol. 68, 585–595. 10.1007/s11103-008-9394-5, PMID: 18781388

[ref51] Rogato A. D’Apuzzo E. Chiurazzi M. (2010). The multiple plant response to high ammonium conditions. The *Lotus japonicus* AMT1;3 protein acts as a putative transceptor. Plant Signal. Behav. 5, 1584–1586. 10.4161/psb.5.12.13856, PMID: 21150259 PMC3115110

[ref52] Saito H. Oiwawa T. Hamamoto S. Ishimaru Y. Kanamori-Sato M. Sasaki-Sekimoto Y.. (2015). The jasmonate-responsive GTR1 transporter is required for gibberellin-mediated stamen development in *Arabidopsis*. Nat. Commun. 6, 6095–7006. 10.1038/ncomms7095, PMID: 25648767 PMC4347201

[ref53] Santi C. von Groll U. Ribeiro A. Chiurazzi M. Auguy F. Bogusz D.. (2003). Comparison of nodule induction in legume and actinorhizal symbioses: the induction of actinorhizal nodules does not involve ENOD40. Mol. Plant-Microbe Interact. 16, 808–816. 10.1094/MPMI.2003.16.9.808, PMID: 12971604

[ref54] Segonzac C. Boyer J. C. Ipotesi E. Szponarski W. Tillard P. Touraine B.. (2007). Nitrate efflux at the root plasma membrane: identification of an *Arabidopsis* excretion transporter. Plant Cell 19, 3760–3777. 10.1105/tpc.106.048173, PMID: 17993627 PMC2174868

[ref55] Serova T. A. Tsyganova A. V. Tikhonovich I. A. Tsyganov V. E. (2019). Gibberellins inhibit nodule senescence and stimulate nodule meristem bifurcation in pea (*Pisum sativum* L.). Front. Plant Sci. 10:285. 10.3389/fpls.2019.00285, PMID: 30930920 PMC6428903

[ref56] Signorelli S. Sainz M. Tabares-da Rosa S. Monza J. (2020). The role of nitric oxide in nitrogen fixation by legumes. Front. Plant Sci. 11:521. 10.3389/fpls.2020.00521, PMID: 32582223 PMC7286274

[ref57] Sol S. Valkov V. T. Rogato A. Noguero M. Gargiulo L. Mele G.. (2019). Disruption of the *Lotus japonicus* transporter LjNPF2.9 increases shoot biomass and nitrate content without affecting symbiotic performances. BMC Plant Biol. 19:380. 10.1186/s12870-019-1978-5, PMID: 31470797 PMC6717371

[ref58] Stougaard J. Abildsten D. Marcker K. A. (1987). The *Agrobacterium* rhizogenes pRi TL-DNA segment as a gene vector system for transformation of plants. Mol. Gen. Genet. 207, 251–255. 10.1007/BF00331586

[ref59] Sun J. Bankston J. R. Payandeh J. Hinds T. R. Zagotta W. N. Zheng N. (2014). Crystal structure of the plant dual-affinity nitrate transporter NRT1.1. Nature 507, 73–77. 10.1038/nature13074, PMID: 24572362 PMC3968801

[ref60] Takanashi K. Takahashi H. Sakurai N. Sugiyama A. Suzuki H. Shibata D.. (2012). Tissue-specific transcriptome analysis in nodules of *Lotus japonicus*. Mol. Plant-Microbe Interact. 25, 869–876. 10.1094/MPMI-01-12-0011-R, PMID: 22432875

[ref77] Tal I. Zhang Y. Jorgensen M. E. Pisanty O. Barbosa I. C. Zourelidou M.. (2016). The Arabidopsis NPF3 protein is a GA transporter. Nat. Comm. 7:11486. 10.1038/ncomms11486 PMC4857387 27139299

[ref61] Tsay Y. F. Chiu C. C. Tsai C. B. Ho C. H. Hsu P. K. (2007). Nitrate transporters and peptide transporters. FEBS Lett. 581, 2290–2300. 10.1016/j.febslet.2007.04.047, PMID: 17481610

[ref62] Tusnàdy G. E. Simon I. (2001). The HMMTOP transmembrane topology prediction server. Bioinformatics 17, 849–850. 10.1093/bioinformatics/17.9.849, PMID: 11590105

[ref63] Urbanski D. F. Małolepszy A. Stougaard J. Andersen S. U. (2012). Genome-wide LORE1 retrotransposon mutagenesis and high-throughput insertion detection in *Lotus japonicus*. Plant J. 69, 731–741. 10.1111/j.1365-313X.2011.04827.x, PMID: 22014280

[ref64] Valkov V. T. Chiurazzi M. (2014). “Nitrate transport and signaling,” in The *Lotus Japonicus* Genome. eds. Tabata S. Stougaard J. (Berlin, Heidelberg: Compendium of Plant Genomes Springer-Verlag), 125–136.

[ref65] Valkov V. T. Rogato A. Alves M. L. Sol S. Noguero M. Léran S.. (2017). LjNPF8.6 controls the N-fixing nodule activity in *Lotus japonicus*. Plant Physiol. 175, 1269–1282. 10.1104/pp.17.01187, PMID: 28931627 PMC5664486

[ref66] Valkov V. T. Sol S. Rogato A. Chiurazzi M. (2020). The functional characterization of LjNRT2.4 indicates a novel, positive role of nitrate for an efficient nodule N_2_-fixation activity. New Phytol. 228, 682–696. 10.1111/nph.16728, PMID: 32542646

[ref67] Villar I. Larrainzar E. Milazzo L. Pérez-Rontomé C. Rubio M. C. Smulevich G.. (2020). A plant gene encoding one-heme and two-heme hemoglobins with extreme reactivities towards diatomic gases and nitrite. Front. Plant Sci. 11:600336. 10.3389/fpls.2020.600336, PMID: 33329665 PMC7710986

[ref68] Wang Y. Y. Cheng Y. H. Chen K. E. Tsay Y. F. (2018). Nitrate transport, signaling, and use efficiency. Annu. Rev. Plant Biol. 69, 85–122. 10.1146/annurev-arplant-042817-040056, PMID: 29570365

[ref69] Wang Q. Huang Y. Ren Z. Zhang X. Ren J. Su J.. (2020). Transfer cells mediate nitrate uptake to control root nodule symbiosis. Nat. Plants 6, 800–808. 10.1038/s41477-020-0683-6, PMID: 32514144

[ref70] Wang C. Yu H. Luo L. Duan L. Cai L. He X.. (2016). Nodules with activated defense 1 is required for maintenance of rhizobial endosymbiosis in *Medicago truncatula*. New Phytol. 212, 176–191. 10.1111/nph.14017, PMID: 27245091

[ref71] Warner C. A. Biedrzycki M. L. Jacobs S. S. Wisser R. J. Caplan J. L. Sherrier D. J. (2014). An optical clearing technique for plant tissues allowing deep imaging and compatible with fluorescence microscopy. Plant Physiol. 166, 1684–1687. 10.1104/pp.114.244673, PMID: 25344504 PMC4256880

[ref72] Waterworth W. M. Bray C. M. (2006). Enigma variations for peptides and their transporters in higher plants. Ann. Bot. 98, 1–8. 10.1093/aob/mcl099, PMID: 16735405 PMC2803549

[ref73] Weber E. Engler C. Gruetzner R. Werner S. Marillonnet S. (2011). A modular cloning system for standardized assembly of multigene constructs. PLoS One 6:e16765. 10.1371/journal.pone.0016765, PMID: 21364738 PMC3041749

[ref74] Williams L. Miller A. (2001). Transporters responsible for the uptake and partitioning of nitrogenous solutes. Annu. Rev. Plant Physiol. Plant Mol. Biol. 52, 659–688. 10.1146/annurev.arplant.52.1.659, PMID: 11337412

[ref75] Witty J. F. Minchin F. R. (1998). Hydrogen measurements provide direct evidence for a variable physical barrier to gas diffusion in legume nodules. J. Exp. Bot. 49, 1015–1020. 10.1093/jxb/49.323.1015

