# Supplementary figures and images for: The Lotus japonicus NPF3.1 Is a Nodule-Induced Gene That Plays a Positive Role in Nodule Functioning
Source: Front Plant Sci. 2021 Jun 18;12:688187. doi: 10.3389/fpls.2021.688187 (PMC8253256; doi:10.3389/fpls.2021.688187)

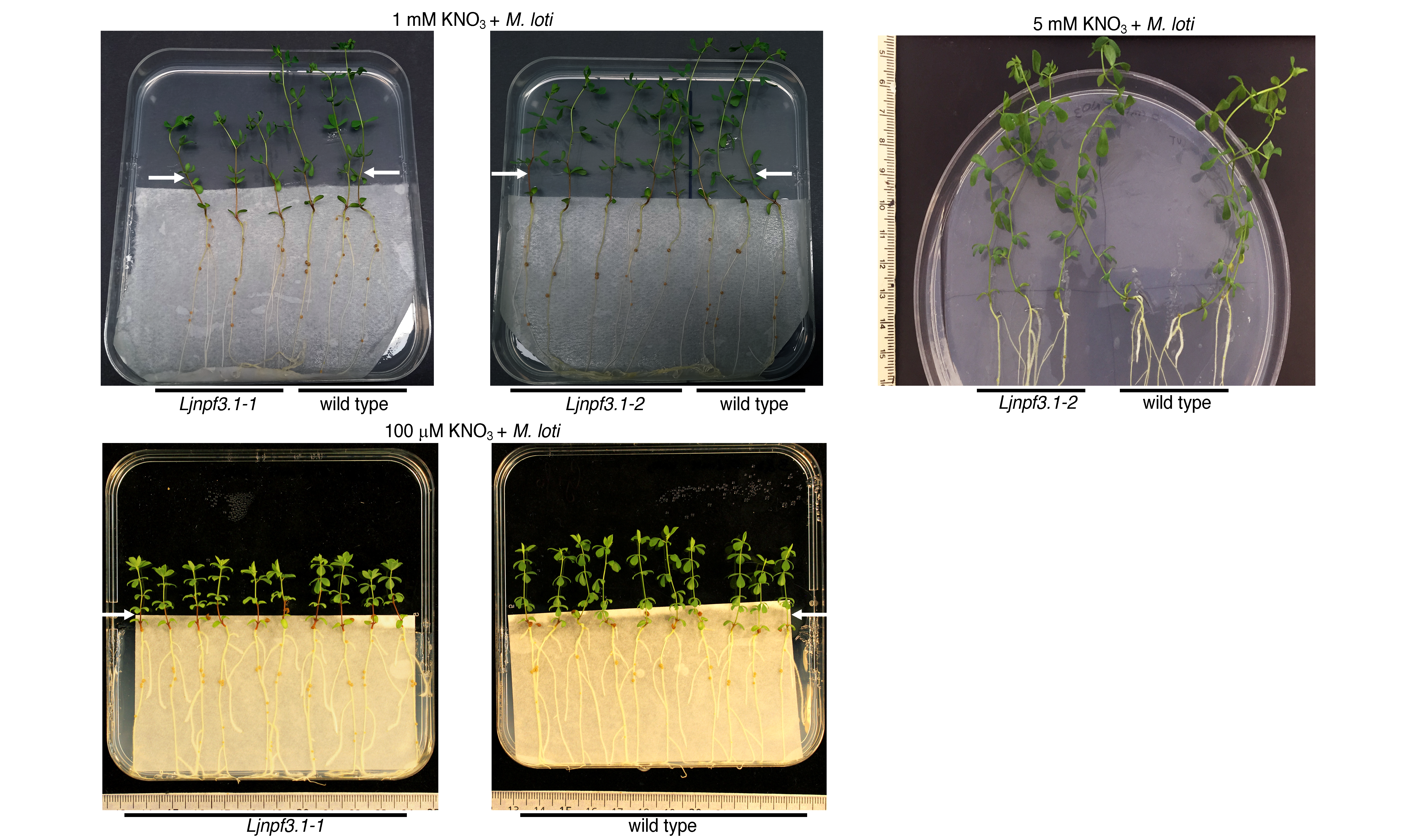

Supplement: Supplementary Figure S1 — Representative images of the wild-type and Ljnpf3.1 plants at 4 weeks after inoculation. Nitrate conditions and plant genotypes are indicated. The white arrows indicate the zones displaying the evident accumulation of anthocyanin in the stems of the mutant plants as compared to the wild-type. [file Image_1.TIFF]

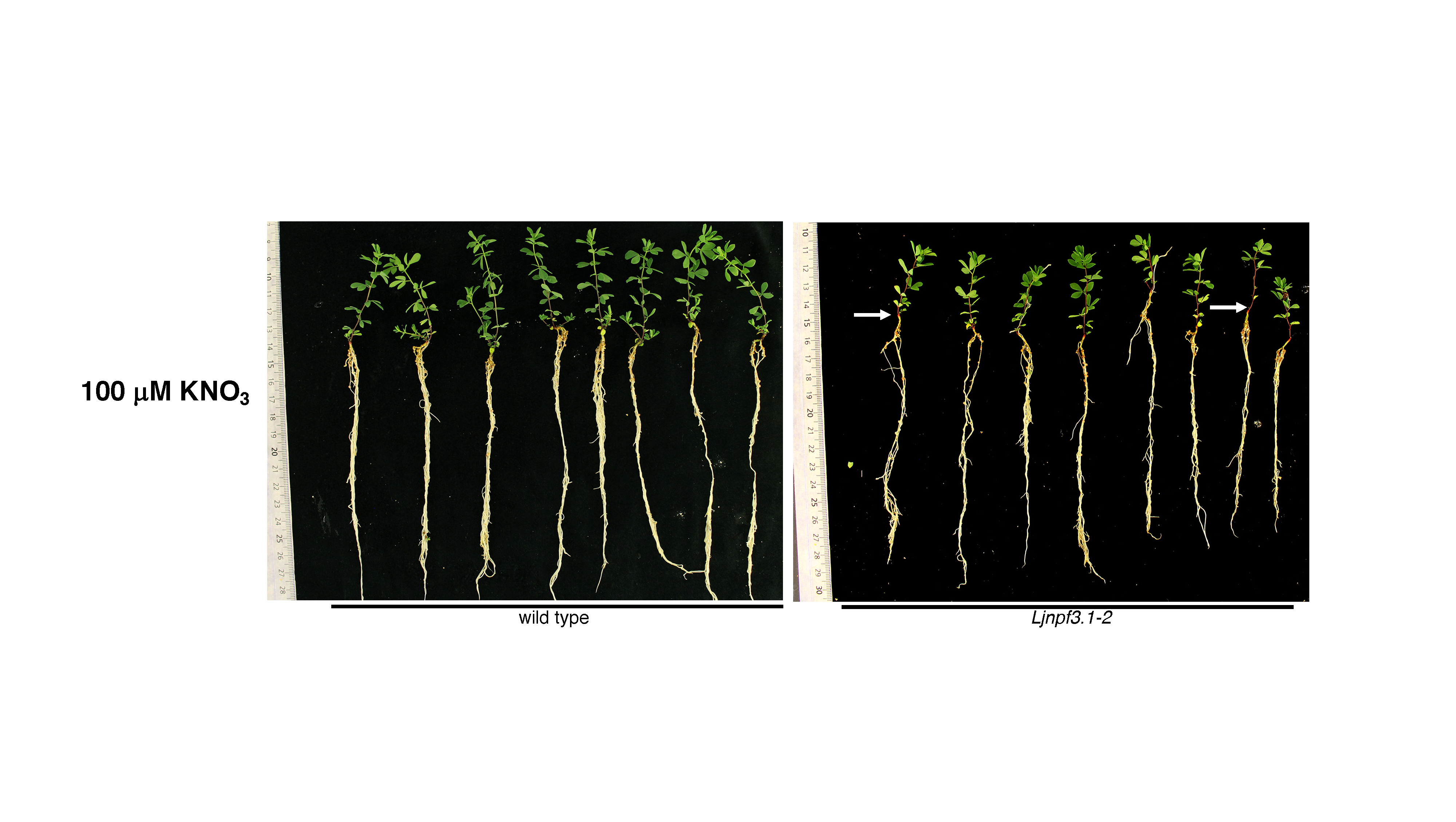

Supplement: Supplementary Figure S2 — Representative images of the wild-type and Ljnpf3.1 plants at 6 weeks after inoculation. Nitrate condition and plant genotypes are indicated. The white arrows are pointed on stems of mutants displaying evident accumulation of anthocyanin. [file Image_2.TIFF]

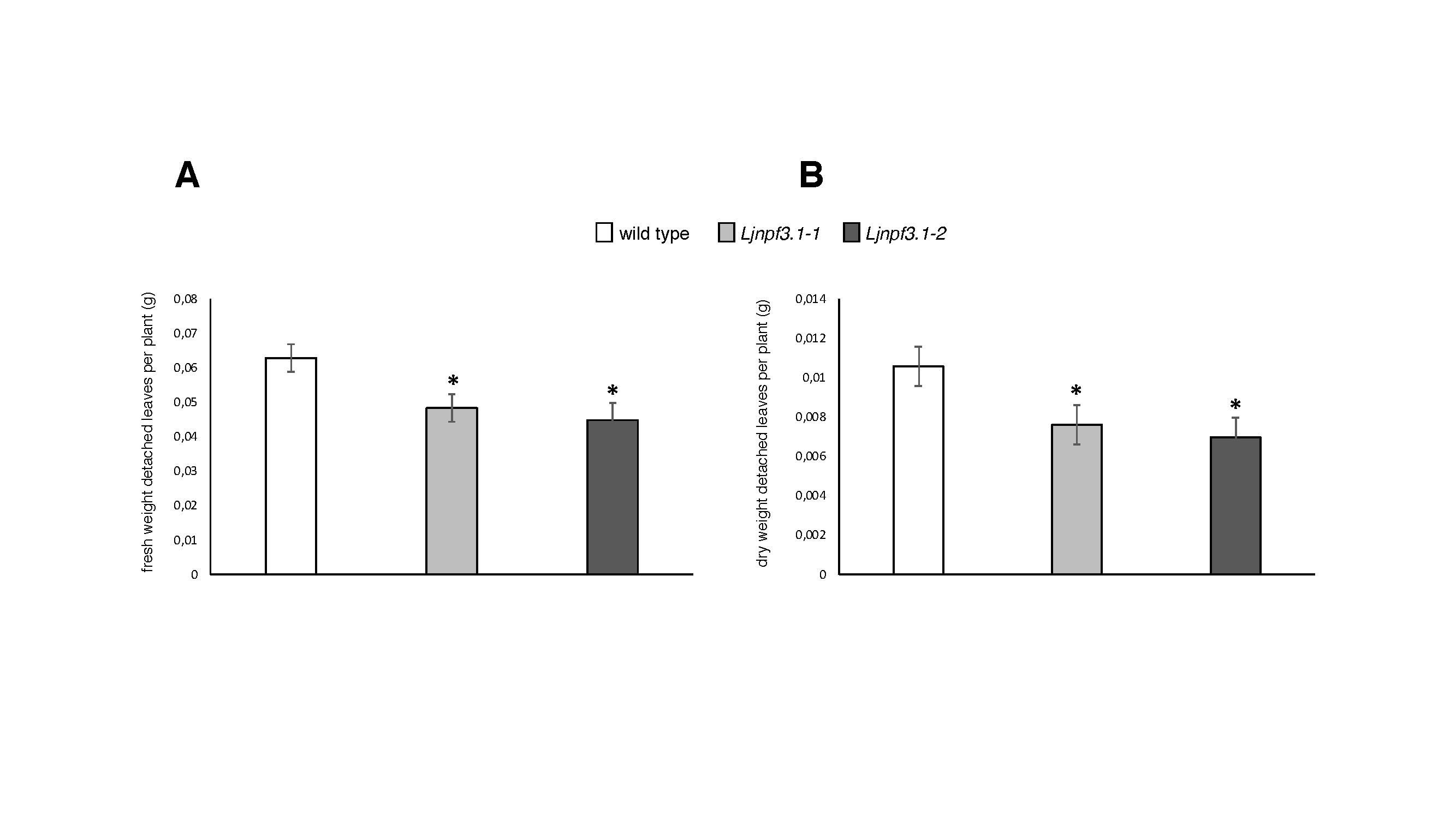

Supplement: Supplementary Figure S3 — Phenotypic characterization of Ljnpf3.1-1 and Ljnpf3.1-2 mutants. (A) Fresh weight of detached leaves. (B) The dry weight of detached leaves. Plants were grown in Petri dishes, in the presence of 1 mM KNO3. Leaves from single plants were detached and immediately weighted at 4 weeks after inoculation. Bars represent the means and SE of measures from three experiments (12 plants per experiment). Asterisks indicate significant differences with wild-type levels. *p < 0.05. [file Image_3.TIFF]
